# Why Hearing Aids Fail and How to Solve This

**DOI:** 10.3389/fnetp.2022.868470

**Published:** 2022-04-25

**Authors:** Ruedi Stoop

**Affiliations:** Institute of Neuroinformatics, University and ETH of Zürich, Zurich, Switzerland

**Keywords:** hearing network physiology, hearing aids, listening process, source separation, cochlear embedding, hair cells innervation

## Abstract

Hearing is one of the human’s foremost sensors; being able to hear again after suffering from a hearing loss is a great achievement, under all circumstances. However, in the long run, users of present-day hearing aids and cochlear implants are generally only halfway satisfied with what the commercial side offers. We demonstrate here that this is due to the failure of a full integration of these devices into the human physiological circuitry. Important parts of the hearing network that remain unestablished are the efferent connections to the cochlea, which strongly affects the faculty of listening. The latter provides the base for coping with the so-called cocktail party problem, or for a full enjoyment of multi-instrumental musical plays. While nature clearly points at how this could be remedied, to achieve this technologically will require the use of advanced high-precision electrodes and high-precision surgery, as we outline here. Corresponding efforts must be pushed forward by coordinated efforts from the side of science, as the commercial players in the field of hearing aids cannot be expected to have a substantial interest in advancements into this direction.

## Introduction

Comparing vision with hearing demonstrates that humans depend as much on auditory as on visual inputs. The compensation of corresponding sensory deficits is, at least in milder cases, much simpler and more successful in the visual domain (glasses and lenses) than in audition.

**FIGURE 1:**
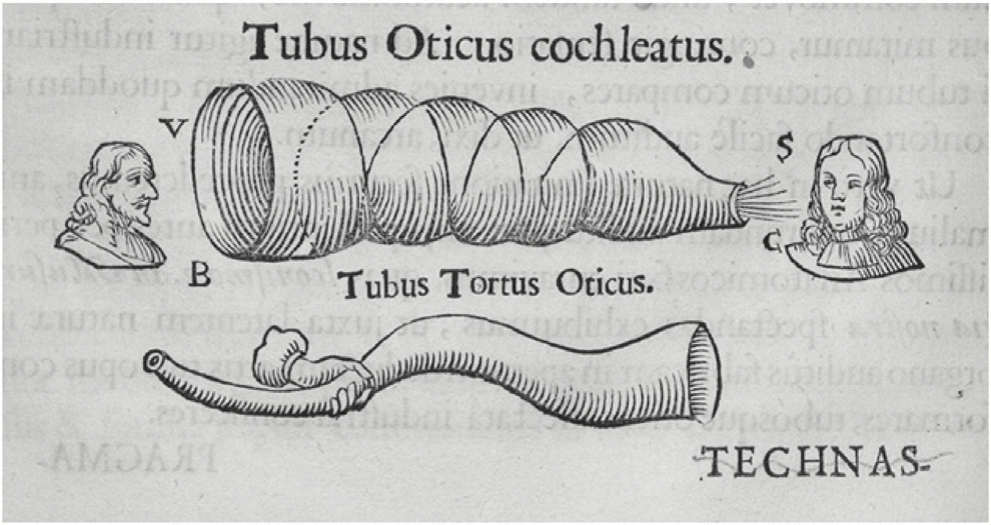
Horns used as hearing devices. From (Kircher, 1673).

**FIGURE 2:**
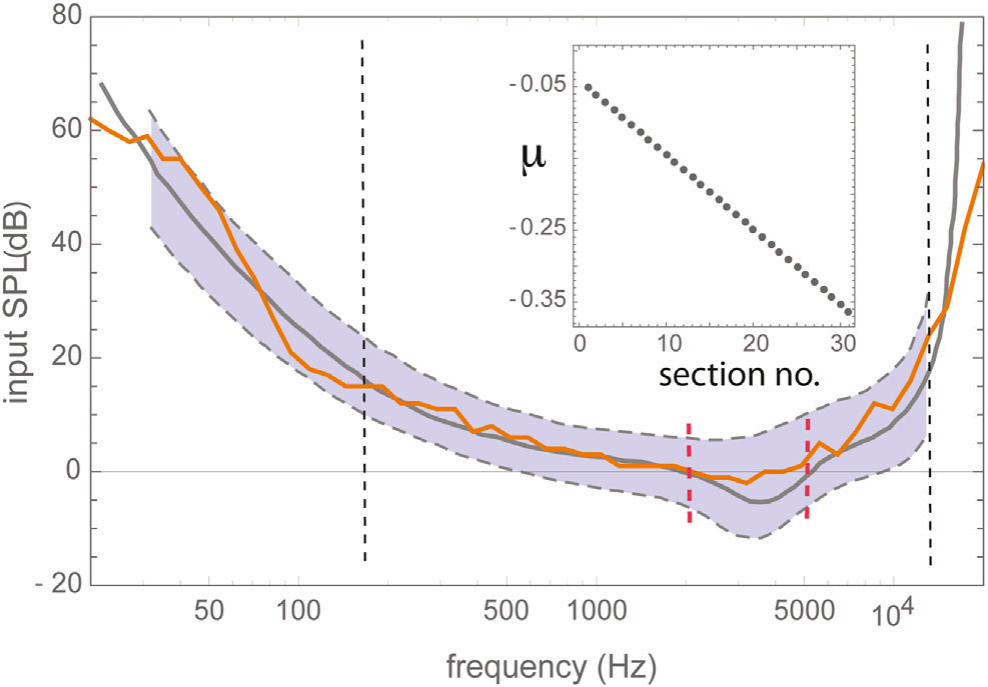
Human hearing threshold. Black dashed vertical lines delimit the proper frequency range of the Hopf cochlea model composed of 30 discretized sections of excitabilities (“Hopf parameters”) μ, centered around decaying center frequencies CF (cf. text references for the model); red vertical lines delimit the area of outer ear influence. Gray curves: data from Zwicker's publication Zwicker and Heinz, 1955, extrapolations thereof dashed. Gray shading: observed human variability. Adapted from (Kanders et al., 2017).

To improve hearing, in extension of the hollow hand, humans used early on animal horns and hallow bones, cf. Figure 1. Later on, these solutions were succeeded by more efficient ear trumpets, made of metal. All of these remedies worked via the bundling of the arriving sound waves at the level of the outer ear. As is shown by the limited influence of the outer ear on the hearing threshold, see Figure 2, this strategy is able to contribute to speech and sound intelligibility only to a limited extent.

Whereas weakness in visual focusing can be compensated by optical glasses, a convincing idea of how to cope with corresponding problems in the auditory field seems to be still missing. Present outer-ear artificial hearing aids, as well as inner-ear cochlear implants, have not yet made any serious attempt to restore the active processes that are naturally involved in biological hearing. Digital hearing aids use computer chip technology to convert sound waves into digital signals, which opens the road to complex input signal processing. As most hearing aid users value speech intelligibility as their top priority, speech enhancers, directional microphones, and noise and feedback cancellation come standard with top-of-the-line hearing aids, helping their users to hear speech even in loud and noisy environments. Among the biggest achievements presented, they process auditory signals according to input volume: Soft speech is made audible, while loud speech is kept comfortable. These implemented capabilities are mostly helpful if an intrinsically useful signal (e.g., speech) is to be separated from an intrinsically un-useful disturber (e.g., noise). As soon as we deal with several signals of potential similar importance (e.g., two speakers), the task becomes a difficult one. Regarding a separation of individual voices from a mixture of voices (the so-called cocktail problem), as well as regarding the perception of music, the results remain to be rather limited, if compared to the human faculty.

## EMOCS at the Heart of Artificial Hearing Aids Problems

**FIGURE 3:**
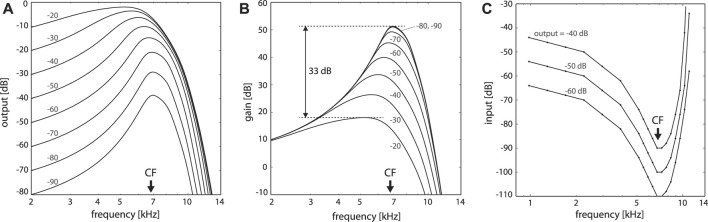
Close-to-biology small signal amplifier implementation, including subcritical tuning and influence of cochlear fluid (endolymph). Hopf cochlea covering 14.08–0.44 kHz with 20 sections; output at Section 5 (CF = 6.79 kHz), stimulation by pure tones. Numbers denote input levels in dB; CF: section’s central frequency. **(A)** Response in dB, **(B)** gain in dB; a difference of 33 dB in peak gain for two input levels differing by 70 dB corresponds to observations in chinchilla (between 20 and 90 dB SPL curves, 32.5 + dB (Ruggero et al., 1997)). **(C)** Tuning curves for fixed output levels. Numbers denote input levels in dB; CF: section’s central frequency.

The heart of the problem that prohibits hearing aids from making more substantial progress comes from two sides. The most advanced hearing aids boast that, in contrast to competitors, they start to distort by their processing input only above 113 dB SPL “Combining this with a music program that allows as little interaction as possible from the more advanced features in the hearing aid, this higher maximum input level allows musicians to enjoy music even in one of these sophisticated hearing aids” (University of Colorado at, 2017; Croghan et al., 2014). This hints at that digital filter and amplification approaches applied to the input signal are too complicated, and inefficient. For digital implementations of the correctly understood nature of the inner ear (the cochlea), neither this (see Figures 2, 3), nor the reproduction of all salient features of human “psychoacoustic” hearing, are difficult tasks (cf. Kern, 2003; Kern and Stoop, 2003; Stoop and Kern, 2004a; Stoop and Kern, 2004b; Martignoli and Stoop, 2010; Gomez and Stoop, 2014; Kanders et al., 2017).

As such a sensor can easily be combined with present-day cochlear implant stimulation electrodes, the real challenge to be solved is the cocktail-party problem. In current hearing aid technology, directional microphones are still the top-notch solution for this problem, although in the meantime methods that use the properties of the sources have been demonstrated to work extremely well (but come with some computational demand). In one of these approaches (Kern and Stoop, 2011), a speaker’s main sound properties are extracted using wavelet-like filters and adapted to changing speech by the “matching pursuit approach”. The work showed that deterministic signal features can be exploited for signal separation of several voices under quite general conditions, which reaches far beyond the noise-speech issue handled by current-day hearing aids (Author Anonyms, 2022).

**FIGURE 4:**
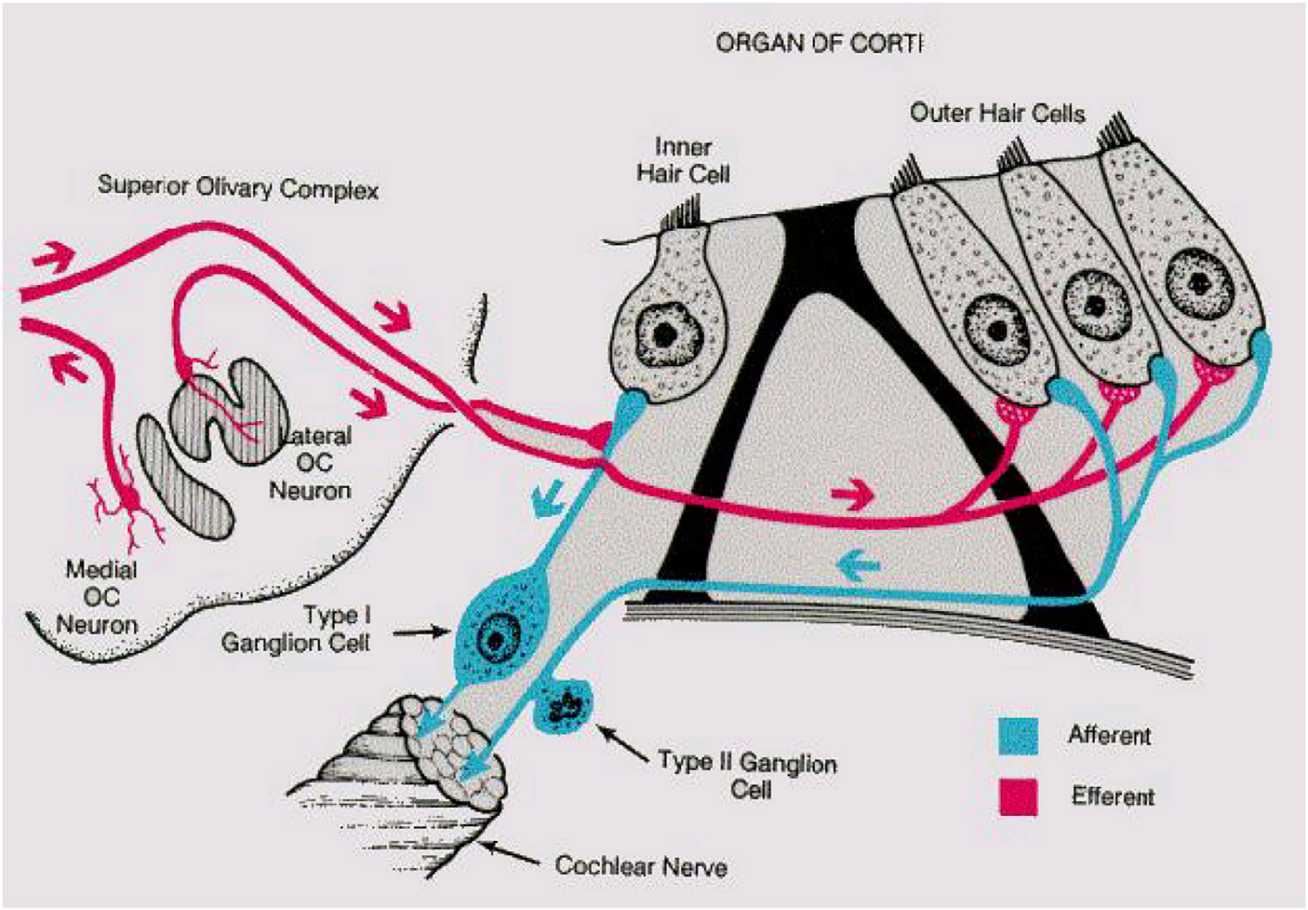
Mammalian listening circuit. Adapted from (Spoendlin, 1966b).

The core of this approach was borrowed from how mammalian (and to a large extent more general: animal) hearing is embedded into feedback loops telling the hearing sensor what to listen to. The lack of an appropriate embedding of the hearing aids into the natural sensory network physiology is the main obstacle against arriving at better hearing aids. The cochlea, the mammalian hearing sensor, hosts in the case of humans at birth, on the order of 3,500 inner hair cells and 12,000 outer hair cells. Outer hair cells amplify the shallow fluid surface waves into which the hearing sensor has converted arial sounds after their arrival at the cochlea (Kern, 2003; Kern and Stoop, 2003). Unfortunately, ageing and inflammatory processes strongly affect—almost exclusively—the outer hair cells in an irreversible manner; the inner hair cells that pick up the amplified wave signals and relay them onwards to the cortex, remain generally unaffected. Biological studies have established that hearing is additionally embedded into several neural loops. In the past, this has been described to great physiological details in the works of Spoendlin (Spoendlin, 1966a; Spoendlin, 1966b), see Figure 4. Most of the current-day’s drawings of the cochlear innervation details are based, often without mentioning, on that work.

From these and ensuing physiological studies on, the purpose of the eminent innervation of the organ of Corti by means of efferent, almost exclusively inhibitory, connections has remained an open problem. The general belief was that they could play a role in the coding of the sound towards neural signals, cf. (Spoendlin, 1966b). The main argument for the particular innervation by efferent cochlear neurons appears to have been that (quotes from Ref. (Spoendlin, 1966b)) “a great number of even small inputs could generate and action potential at the initial segment,” “the role of the efferent fibres for the coding of the acoustic message at the level of the organ of Corti is not yet entirely understood. The only directly demonstrated action is an inhibitory effect on the afferent nerve impulses. This inhibition is, however, not very strong and it is hard to believe that such ab extensive efferent innervation system in the cochlear receptor would have only such a limited function. It is more likely that the efferent fibres have a much more complex function than this relatively restricted inhibition which can be measured. They might influence the afferent impulses in a more qualitative than quantitative fashion,” “Many different phenomena of the auditory physiology might depend on the efferent innervation of the cochlea. However, only a few have been directly demonstrated hitherto as for the adaptation phenomenon,” “As long as these coding mechanisms can not be simulated, an artificial cochlea will only provide a very rudimentary function”. After Spoendlin, this view persisted, in essence, until today.

## How EMOCS Work

**FIGURE 5:**
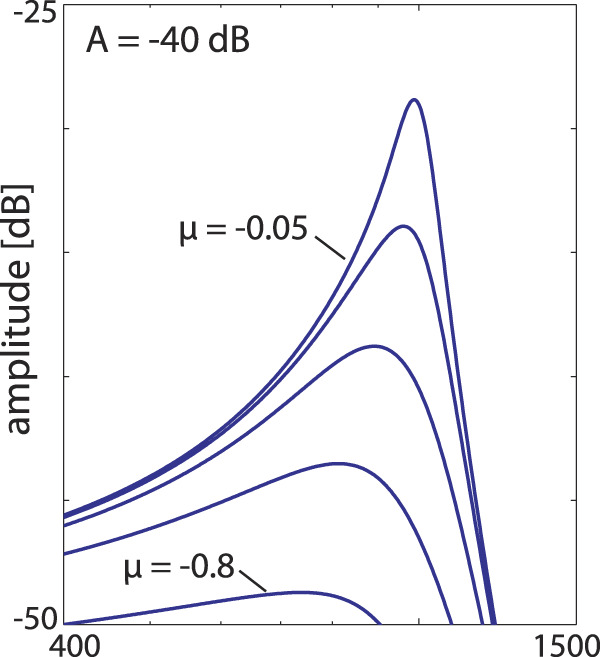
Single Hopf amplifier response (Kern, 2003; Kern and Stoop, 2003), describing the behaviour of outer hair cells with a preferred frequency CF embedded into the basilar membrane. Frequency selectivity regarding different distances *μ* ∈ { − 0.05, − 0.1, − 0.2, − 0.4, − 0.8} from bifurcation point (showing slightly more asymmetry if compared to Figure 3).

In our view, these loops, however, foster the biological need of mammals to identify a large spectrum of pre-defined relevant signals as follows. Mathematically, the properties of the outer hair-cells can be represented by dynamical systems that are close to a Hopf bifurcation and act as small-signal amplifiers (Derighetti et al., 1985; Wiesenfeld and McNamara, 1985; Wiesenfeld and McNamara, 1986; Camalet et al., 2000; Eguíluz et al., 2000; Duke and Jülicher, 2003; Kern, 2003; Kern and Stoop, 2003; Magnasco, 2003). Systems close to a Hopf bifurcation not only depend on the input strength and frequency of the stimulating signal, but also on how close the system actually is to the Hopf bifurcation (whereas in the concurrent approach of Refs. (Eguíluz et al., 2000; Magnasco, 2003), this point was left open, in Refs. (Camalet et al., 2000; Duke and Jülicher, 2003), systems were required to be poised exactly at the bifurcation point). The distance to the bifurcation point is the main element that rules the amplifier’s specificity, see Figure 5. Moreover, this feature provides the access point for the embedding of the sensor into the physiological network.

**FIGURE 6:**
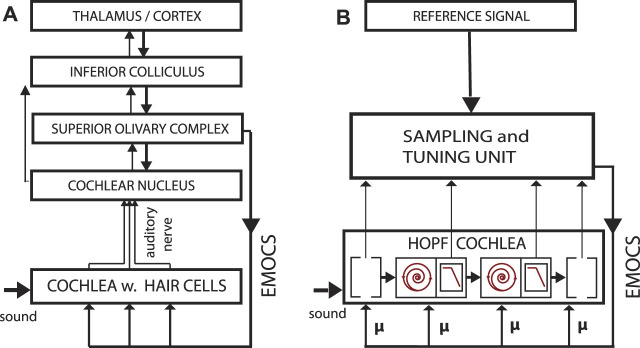
**(A)** Biological vs. **(B)** artificial implementation of the hearing—listening circuit. Listening is a dedicated activity that requests and represents a particular computational effort, involving “EMOCS” (efferent medial olivocochlear stimulations), adapted from (Kern and Stoop, 2011).

**FIGURE 7:**
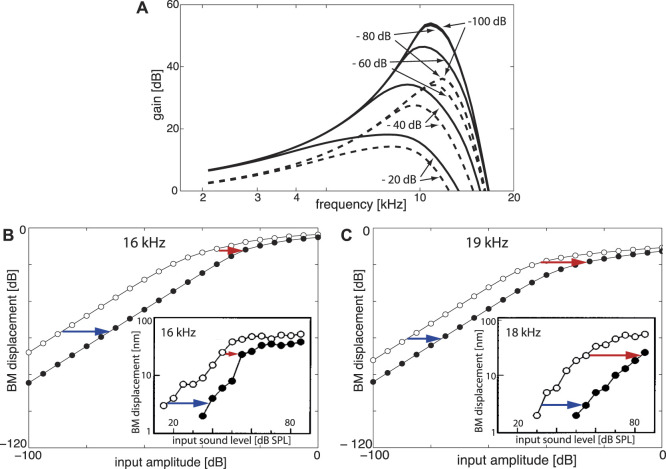
EMOCS effects: **(A)** Gain isointensity curves at Section 5 (*f*_*ch*_ = 1.42 kHz) without (solid lines) and with (dashed lines) EMOC input. From flat tuning (*μ* = −0.1 for all sections, EMOC stimulation is implemented by shifting to *μ*_5_ = −1.0 ( − 80 and − 100 dB lines collapse). **(B)** Upon 16 and **(C)** 19 kHz pure tone EMOCS, implemented by a shift from a flat tuned cochlea from *μ*_2_ = −0.05 to *μ*_2_ = −0.5, BM levels at Section 2 (*f*_*ch*_ = 16.99 kHz) shift from open circles to full circles. Insets: Corresponding animal experiments (Russell and Murugasu, 1997).

There is strong biological evidence that EMOCS (efferent medial olivocochlear stimulations) regulate the Hopf elements’ distances to the bifurcation point, see Figure 6, most likely in a stronger manner than originally anticipated by Spoendlin. Activated EMOCS drive the system away from the bifurcation point, exerting in this way an inhibitory effect on the targeted Hopf amplifiers, see Figure 7. Changed individual amplification entrains striking effects at the level of the whole sensory organ. A recent work (Stoop and Gomez, 2016) focused on the excitation network from the nonlinear interaction of excited amplifiers generating combination tones. It was shown that under absence of EMOCS, signals of two complex tones of random amplitude and frequency, generated “activation networks” with the size *s* of links being distributed according the typical critical branching network paradigm (exponent *a* = 3/2, at − 60 dB input, the typical strength of human speech). Stronger input ( − 50 dB) yields distributions that are typical for supercritical states, whereas upon EMOC activation, the distributions change into a subcritical shape. This reveals that at the most relevant working condition, the system’s information-receiving predisposition is at criticality, whereas listening (implemented by means of EMOCS) is characterized by subcritical states (Lorimer et al., 2015).

## Listening to Sounds

**FIGURE 8:**
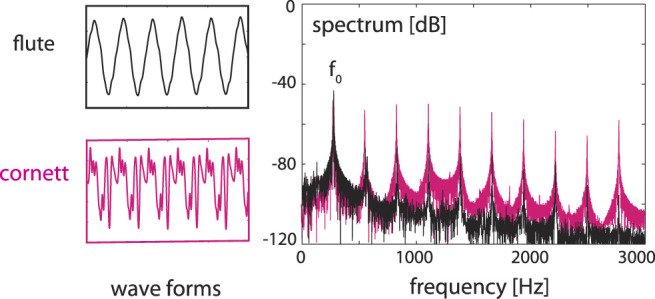
Sounds of a cornett and a flute (left) at the same fundamental frequency *f*_0_, superimposed (right), static case.

In the following we demonstrate how EMOCS represent the faculty of listening at the biological level. For this, we reinterpret earlier results (Gomez et al., 2014) on sound separation from mixtures of sounds, see Figure 8.

To achieve separation, the listening process fosters a previously identified subset of amplifiers, disposing amplifiers that are not associated with the desired signal. This process is implemented in biology with the help of information from the brain, mediated by means of EMOCS: Nerves leading from the brain to the cochlea via medial olivocochlear stimulation suppress the efficacy of the targeted cochlea sections by, technically speaking, pushing corresponding Hopf amplifiers further away from their point of bifurcation, cf. Figure 4. The correctness of this translation from biology to the model has, we re-iterate, been fully corroborated by the available data from the biological effects by EMOCS in Figure 7.

**FIGURE 9:**
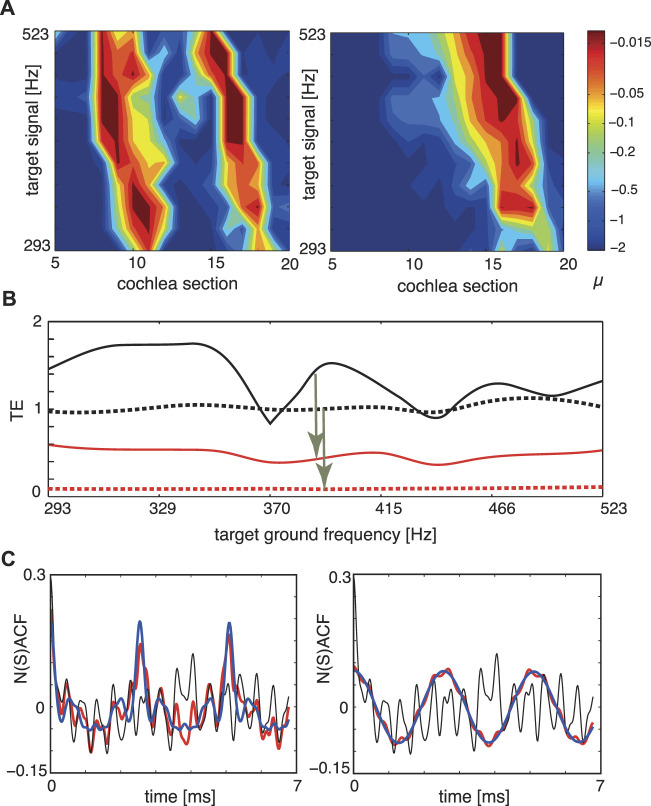
Sound separation, dynamic case, where the target instrument changes the height of the generated tone: **(A)** Tuning patterns, dynamical case. Colors indicate the Hopf parameter values of the sections. Left: Cornett vs. flute (disturber). Right: Flute vs. cornett (disturber). **(B)** TE for the two target signals of **(A)**. Flat tuning: black; *μ*-tuning: red. Full: cornett target, dashed: flute target. Arrows indicate improvements by EMOCS. **(C)** NSACF, NACF for the two target signals of **(A,B)** at a chosen target ground frequency. Flat tuning: black, *μ*-tuning: red, target signal: blue. Targets at 392 Hz, disturbers at 2,216 Hz. After Ref. (Gomez et al., 2014).

**FIGURE 10:**
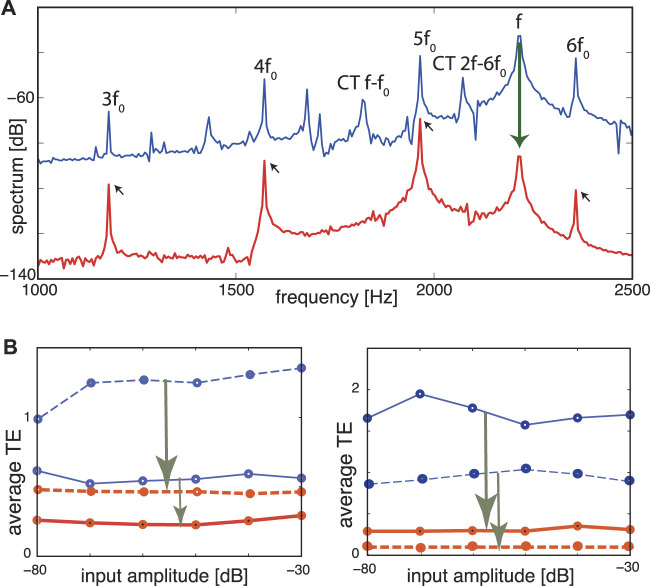
TE improvement by *μ*-tuning, static case. **(A)** Frequency spectrum at Section 8 (*CF* = 1964 Hz). Blue: Flat tuning (−80 dB, target cornett *f*_0_ = 392 Hz, disturber flute *f* = 2,216 Hz). Cross-combination tones (CT, two explicitly labeled) between the flute’s fundamental *f* and higher harmonics of the cornett are clearly visible. Red: Optimized tuning. *f* (flute) and cross-combination frequencies are suppressed, leaving a harmonic series of the target (small arrows). **(B)** Averaged TE over 13 different fundamental target frequencies (steps of 1 semitone) demonstrates input amplitude independence. Blue lines: flat tuning. Red lines: optimized *μ*-tuning. Left panel: (full lines) target sound cornett (277–554 Hz), disturbing sound flute (at 277 Hz); (dashed lines) same target but flute at 2,216 Hz. Right panel: same experiment with target and disturber interchanged. TE improvements: arrows in **(B)**. From (Gomez et al., 2014).

In Figure 9 we report the result of our biomorphic implementation of the listening process, where in subpanel a) we show how the tuning of the amplifiers changes, as the target object, the musical organ, increases its fundamental frequency in time. To assess how close we arrive to the target, we use our tuning error measure *TE* that has the expression $TE\left(x,y\right)=\frac{\|norm.\left(\sum_{i}ACF\left(f_{i}\left(x+y\right)\right)\right)-NACF\left(x\right){\|}_{2}}{\|norm.\left(\sum_{i}ACF\left(f_{i}\left(x+y\right)\right)\right)-NACF\left(y\right){\|}_{2}},$ where *f*_*i*_ denotes the output at section *i* of the cochlea and the summations extend over the *N* sections. NACF is the full normalized summary autocorrelation function accounting for all sound characteristics such as e.g., timbre); to measure how strongly a mixture of two input sounds *x*, *y*, is biased towards component *x*, we use the Euclidean distance between the full mixture’s NACF (“NSACF”)and the target signal *x*’s NACF, divided by the Euclidean distance between the full mixture’s NACF (“NSACF”) and the undesired signal *y*’s NACF. TE values are between 0 and *∞*, where *TE* = 0 indicates a perfect focus, and a larger TE is a less successful target focus. If one source dominates the mixture, then even before tuning, TE values below unity may emerge. The results displayed in panel b) demonstrate the implemented processes’ efficacy also for time-varying target signals. For fixed target ground signals, panel c) demonstrates how close we get in terms of NACF (red) to the target signals (blue). More information can be extracted by scrutinizing the static case at the level of the signals’ spectra, evaluated at variable signal strengths, which exhibits how EMOCS succeed in suppressing in particular the combination tones among the two signals, see Figure 10A). Also upon a variation of the input amplitudes of the two signals, the tuning errors remain small (see Figure 10B).

**FIGURE 11:**
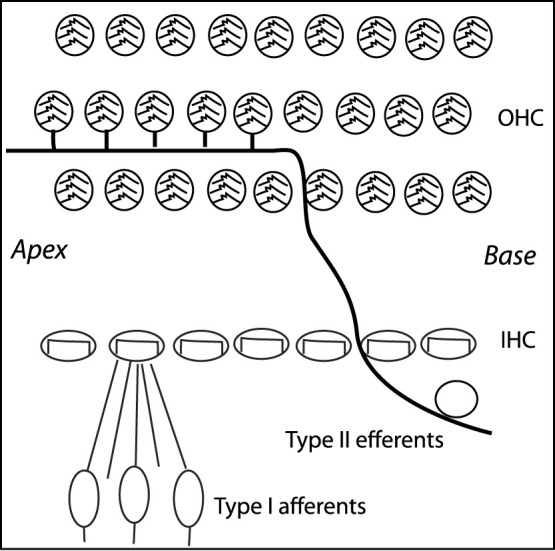
Cochlear hair-cells innervation.

**FIGURE 12:**
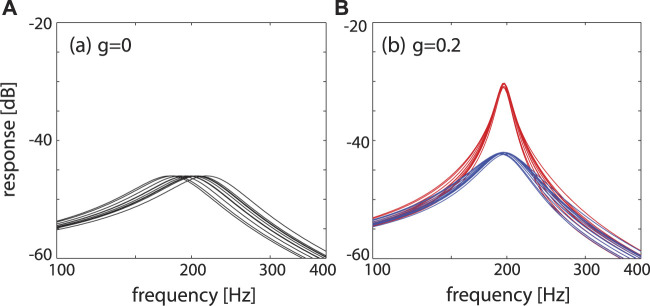
Effects of signal coupling. Response of *N* = 10 systems, characteristic frequencies distributed around 200 Hz, to a test signal of amplitude − 60 dB. **(A)** Uncoupled, *μ* = −0.2, **(B)** signal-coupled (blue: *μ* = −0.3, red: *μ* = −0.2), exhibiting a coherent and sharply tuned response around *f*_*c*_ ≃ 200 Hz. From (Gomez et al., 2016).

A second principle that comes to the aid of the EMOCS is the particular efferent innervation within the cochlea, see Figure 11. The particular arrangement of the innervation of out hair cells expresses a feedforward signal-coupling scheme that has been analyzed in Ref. (Gomez et al., 2016), with the result that such an arrangement fosters a collective signal-sharping, as is demonstrated in Figure 12. Such a signal sharpening may explain the surprising frequency discrimination (Spoendlin, 1966b) of the mammalian hearing sensor. To exhibit this, we consider two frequency-rescaled unforced Hopf systems that interact via their output signals (i.e., perform a “signal-coupling”), which yields $\frac{d}{dt}z_{1}=\omega_{1}\left(\mu +i\right)z_{1}-|z_{1}{|}^{2}z_{1}+\frac{g_{21}}{2}z_{2}$ , $\frac{d}{dt}z_{2}=\omega_{2}\left(\mu +i\right)z_{2}-|z_{2}{|}^{2}z_{2}+\frac{g_{12}}{2}z_{1}$ , where *ω*_1_, *ω*_2_ are the characteristic frequencies of the systems and *g*_*ij*_ captures the coupling from system *i* to system *j*; the factor of 1/2 facilitates the generalisation to the *N* systems used for Figure 12, where we can see that signal-coupling leads to a sharpening of the response. In our biomorphic model, the corresponding signal sharpening is comprised in the degree of compartimalization of the cochlea combined with appropriately chosen tuning parameters *μ*.

### A Program for Solving the Listening Problem

**FIGURE 13:**
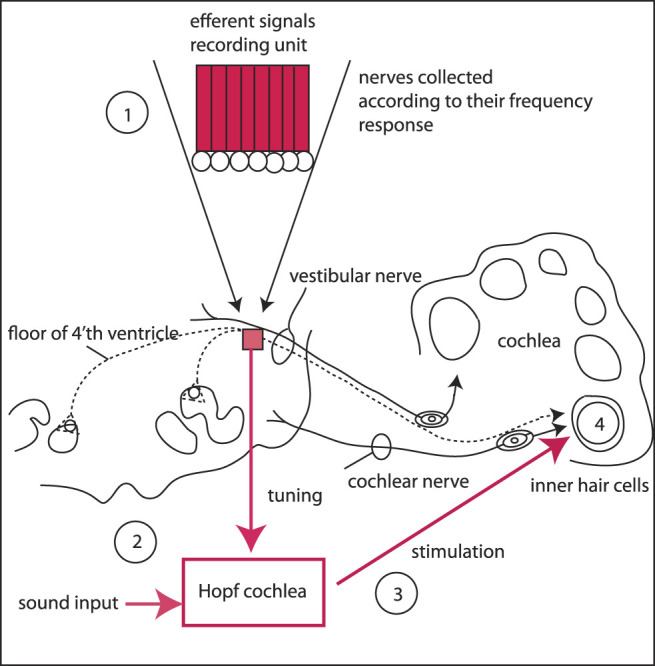
Proposed solution of the listening problem: Using a recording array for the efferent signals to the cochlea (1), the Hopf cochlea (2) is tuned away from the “flat” (i.e., normally distributed *μ*'s) according to the will of the listener. The tuned amplified signal is then sent to the stimulation electrodes (3) of inner hair cells (4). In the recording unit (1), the efferent nerves are grouped according to their frequency response and subject to the micro-surgical limitations.

For the implementation of the full physiological hearing circuitry, we propose to use a recording array for the efferent signals to the cochlea, that will serve to tune the Hopf cochlea away from the “normally” distributed *μ*'s (that decay slowly along the cochlea, see (Gomez and Stoop, 2014; Kanders et al., 2017)), reflecting in this way the will of the listener to focus on the remaining signal part. In the recording unit, the efferent nerves are grouped according to the intrinsic biological resolution modulo the available micro-surgical possibilities. The grouped signal then modifies the Hopf cochlear amplification such that the input signal to the cochlea is selectively amplified in the described manner, whereupon it is sent towards the stimulation electrodes of the inner hair cells, see Figure 13. With such a setting—that we hope to be feasible in the near future—hearing could become fully restored, reconciling the limitations of present-day hearing aids.

### Conclusion

Our work opens a perspective towards the development of more adequate cochlear implant hearing aids, responding to the listener’s desire for the selection of particular sounds. This is of importance for the cocktail party problem as well as for listening to many-instrumental music. With the recognition of the physiological network that hearing is embedded in, and with the proper re-embedding of the biomorphic Hopf cochlea (Stoop et al., 2008) into this physiological network, we can reach far beyond of what is presently achievable by present-day hearing sensor technology. The task to achieving this in the near future should, however, preferentially not be delegated the hearing-aid industry, as the latter cannot be too much interested in the corresponding shift from digital engineering to high-precision micro-surgery, required for the embedding of the sensor into the physiological network of hearing.

## Data Availability

The original contributions presented in the study are included in the article, further inquiries can be directed to the corresponding author.
